# Finding Alternatives to the Dogma of Power Based Sample Size Calculation: Is a Fixed Sample Size Prospective Meta-Experiment a Potential Alternative?

**DOI:** 10.1371/journal.pone.0158604

**Published:** 2016-06-30

**Authors:** Elsa Tavernier, Ludovic Trinquart, Bruno Giraudeau

**Affiliations:** 1 INSERM, U1153, Paris, France; 2 CHRU, Tours, France; 3 INSERM CIC 1415, Tours, France; 4 Department of Epidemiology, Mailman School of Public Health, Columbia University, New York, New York, United States of America; 5 Université François-Rabelais de Tours, Tours, France; University of British Columbia, CANADA

## Abstract

Sample sizes for randomized controlled trials are typically based on power calculations. They require us to specify values for parameters such as the treatment effect, which is often difficult because we lack sufficient prior information. The objective of this paper is to provide an alternative design which circumvents the need for sample size calculation. In a simulation study, we compared a meta-experiment approach to the classical approach to assess treatment efficacy. The meta-experiment approach involves use of meta-analyzed results from 3 randomized trials of fixed sample size, 100 subjects. The classical approach involves a single randomized trial with the sample size calculated on the basis of an *a priori*-formulated hypothesis. For the sample size calculation in the classical approach, we used observed articles to characterize errors made on the formulated hypothesis. A prospective meta-analysis of data from trials of fixed sample size provided the same precision, power and type I error rate, on average, as the classical approach. The meta-experiment approach may provide an alternative design which does not require a sample size calculation and addresses the essential need for study replication; results may have greater external validity.

## Introduction

Randomized trials are usually planned as a single study, aimed at comparing the effectiveness of two or more treatment groups. Established practice requires investigators to calculate the sample size based on a power calculation. However, these calculations can be challenging. Calculating the sample size to ensure a target power requires at least 1) specifying the treatment effect and 2) specifying a second parameter, such as the event rate in the control arm for binary outcomes, called a nuisance parameter. These should ideally be justified based on previous studies or other evidence [1–3]. However, a review of 446 protocols submitted to UK ethics committees in 2009 found that only 190 (43%) justified the treatment effect used in the sample size calculation, and only 213 (48%) justified the nuisance parameter related to the control group [4]. Another review of 215 articles published in 6 high-impact-factor medical journals found large discrepancies between the pre-specified parameters used in the sample size calculation and those observed after the trial was complete. This can adversely affect study power [5–7].

The first issue with sample-size calculations is that we sometimes lack sufficient information to reliably justify the choices of parameters used in the calculation. Even with information from previous studies, new trials may focus on a population different from that previously studied [5], or the only available data may be of poor quality. In some cases, previously data may not exist.

The second issue is that many researchers do not seem to consider their research as a contribution to the totality of the existing evidence. In the mid- to late-1990s, Cooper et al. [8] observed that even when a Cochrane review was available, the proportion of investigators using it when designing a new trial was low: in a survey of 24 authors, only 2 had used the relevant Cochrane review to design their study. Therefore, convenient information to determine the sample size of a new trial is often wasted.

Finally, such an approach is somewhat idealistic, because it overshadows practical issues such as recruitment and cost. In practice, it is common to readjust the power calculation to obtain a more feasible sample size, a practice Schulz et al. mocked as “sample size samba" [9]. It may even lead to changing the primary outcome to something less relevant, but which leads to a more achievable sample size. Eventually it may prevent the implementation of a trial and lead to abandoning the project.

In the last decade, several authors have expressed the need to be more pragmatic about sample sizes. Norman et al. [10] argued that in the absence of good data, *"it would be better to determine sample size by adopting norms derived from historical data*, *based on large numbers of studies of the same type"*. The authors acknowledged that *"made-to-measure"* calculations can be used if sufficient information is available but encouraged researchers to use *"off-the-peg"* sample sizes otherwise. Bacchetti et al [11] argued that researchers should take into account costs and feasibility when justifying the sample size of their trial. One isolated example is De Groot's trial that studied a rare disease [12]. They determined the sample size by resources rather than statistical considerations.

Simultaneously, Clarke et al [13,14] repeated their call to design and report randomized trials in light of other similar research. They clearly stated that reports of clinical trials should begin and end with up-to-date systematic reviews of other relevant evidence. Although meta-analyses are intrinsically retrospective studies, some authors suggested prospective meta-analyses [15]. Thus, Chalmers et al. encouraged researchers to use information from research currently in progress and to plan collaborative analyses [15], indicating that *“prospectively planned meta-analyses seem likely to offer an important way to generate precise and generalizable estimates of effects”*.

To address the need for pragmatic sample sizes alongside the need to summarize the totality of evidence, we explored the approach of a prospectively planned meta-analysis suggested by Chalmers et al, which we named a “meta-experiment”. The meta-experiment approach would be to plan several fixed-size randomized trials, as a united whole, conducted in parallel in different investigation centers and with a meta-analysis of the results. We investigated when this design could be adopted. We then used a simulation study to compare statistical properties of such an approach to a classical approach based on a single randomized trial with a pre-determined, classically calculated sample size. Our aim was to assess whether the meta-experiment could be statistically efficient, and whether it merits further investigation. This paper first describes the concept of a meta-experiment along with its scope of use, and then displays its statistical properties.

## Materials and Methods

### Meta-experiment

The meta-experiment involves different researchers in separate centers conducting independent trials which address the same question. In practice, any number of separate trials could be conducted, but for the purposes of this paper, we consider 3 researchers from 3 different centers, with each trial using the same fixed sample size of 100 patients. We consider 3 trials because a series of 65 meta-analyses revealed that we need a low number of trials (i.e., about 3 to 5) for the results of a meta-analysis to approach the final pooled value [16]. Furthermore, we assume a fixed sample size of 100 patients for each of the 3 trials, to be feasible in terms of recruitment for a wide range of populations of interest. A sample size of 100 subjects per randomized trial corresponds to the median sample size from a series of 77,237 studies from 1,991 reviews [17]. The primary outcome is pre-specified and clearly stated in the protocols of the 3 trials, to avoid any temptation of data dredging. However, no quantitative hypothesis for the expected treatment effect is made, and no sample size calculation is performed.

#### Scope of use

The meta-experiment design is not intended as a miracle cure for every clinical trial, but as a pragmatic design that would deal with the financial and recruitment difficulties that can occur when running in large trials. The situations where the meta-experiment design could be used are numerous. If we lack sufficient information to conduct a reliable sample size calculation and if a fixed sample size of 100 for the 3 trials is a feasible "off-the-peg" sample size in terms of resources, then the meta-experiment approach could be implemented. The meta-experiment could also be the first step in the assessment of a treatment effect, providing information for the design of a potential further trial to be conducted after the meta-experiment is complete. However, this strategy should not be adopted if we have sufficient information to know that only a very small treatment effect is plausible: then performing 3 trials of sample size 100 each will add little information. In practice, this means that if available analyses have shown that the odds ratio is certainly less than 1.5 it would be better to perform a classical sample size calculation. This strategy should also not be used if recruiting 100 participants is not feasible (e.g. for an extremely rare disease, or a highly expensive treatment). Finally, this strategy should not be used in the opposite situation where it is very easy to recruit a very large number of participants (i.e. 500 participants or more) in a single trial. Indeed the meta-experiment design was initially though in order to face the financial limitation of academic research.

### Simulation study

**Fig 1:**
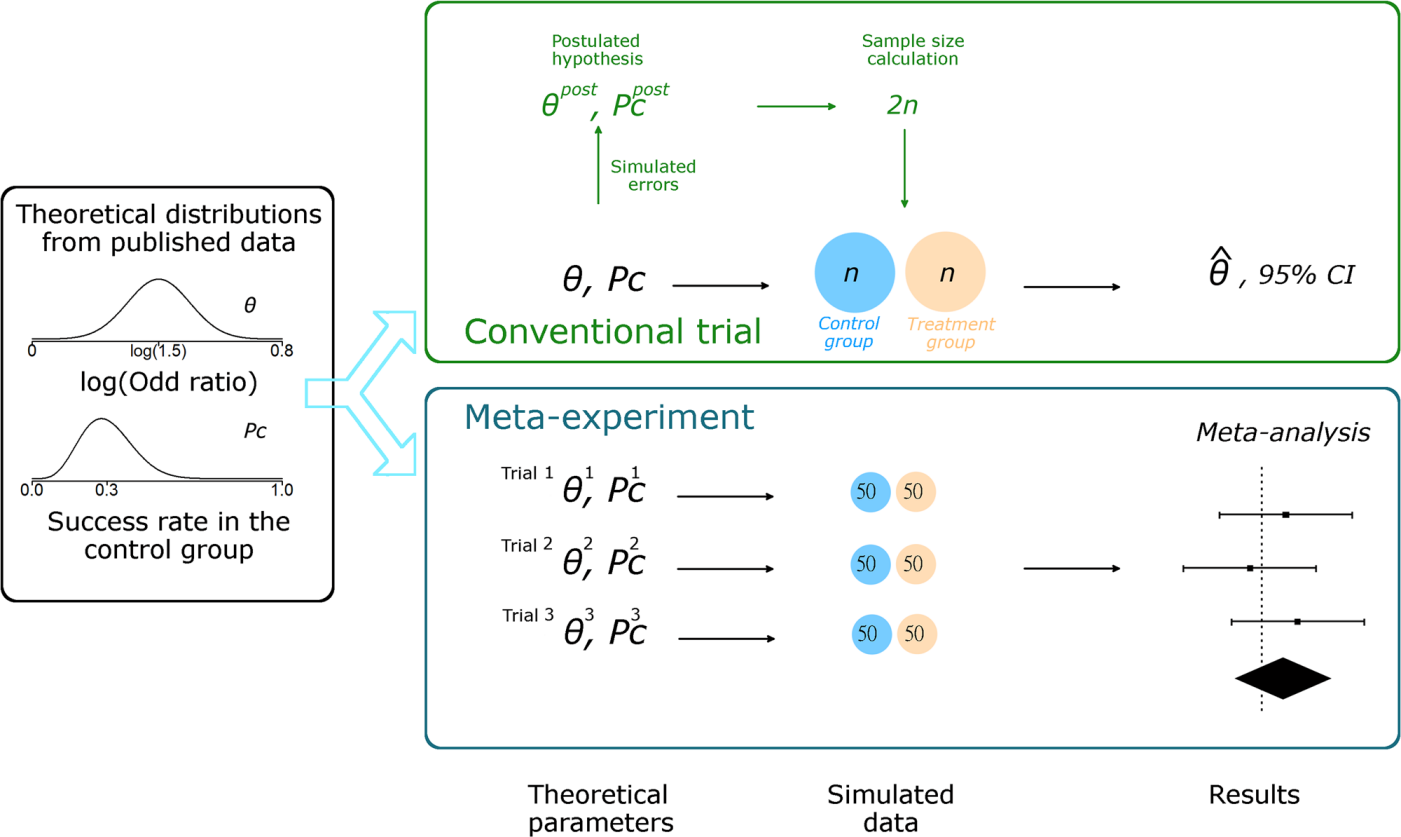
Simulation study with a non-null treatment effect. Theoretical distributions are used to draw the true treatment effects and success rates in the control group for only one trial with the conventional approach and for each of the 3 trials with the meta-experiment. The log odds ratio *θ* is drawn from a normal distribution with mean log(1.5) and SD 0.1. The success rate in the control group *p*_*c*_ is drawn from a beta distribution with mean 30% and SD 10%. With the conventional approach, relative errors are simulated to deduce the postulated hypothesis in designing the trial. The sample size 2n is calculated to ensure 80% power. A trial of size 2n is simulated from the true treatment effect and success rate, and analyzed. With the meta-experiment approach, the same theoretical distributions are used to draw 3 treatments effects *θ*^1^, *θ*^2^ and *θ*^3^, and 3 success rates $p_{C}^{1}{,\text{ p}}_{\text{C}}^{2}$ and $p_{\text{C}}^{3}$. 3 trials with sample size 100 each are simulated from the 3 treatment effects and success rates. Then the data are meta-analyzed in a random-effects model, allowing for variation between the results of the 3 trials.

Our simulation study compared one randomized trial with a classical sample size calculation, for which investigators need to formulate a hypothesis, to a meta experiment of 3 trials with a fixed-size of 100 patients in each (Fig 1). Full details of the algorithms used are in the S1 File.

#### Data simulation and analysis

We considered a two—arm parallel-group framework with a binary outcome. The treatment effect is measured as the logarithm of the odds ratio (OR).

Classical sample size calculation: with the classical approach, the sample size is calculated on the basis of an *a priori*-formulated hypothesis, the randomized trial is conducted, and data are analyzed by a univariate statistical test. The sample size calculation is based on a hypothesized treatment effect and an *a priori*-assumed success rate for the control group. Both parameters are pre-specified and are subject to error. We used data from a previously published review [6] to calibrate these errors. An error for the assumed success rate for the control group and an error for the postulated hypothesized treatment effect were drawn from two normal distributions. Details of the exact parameters are in the S1 File.

Classical approach: we draw a treatment effect value *θ* from the normal distribution of treatment effects. In the situation of a non-null treatment effect, we used a distribution with mean log(1.5). Then we draw a success rate *p*_*c*_ from the Beta distribution. For each of these 2 parameters, we draw errors from the empirical error distributions previously observed. Combining the values drawn from the theoretical probability distribution and their associated errors, we derived an *a priori*-hypothesized treatment effect (denoted *θ*^post^, a postulated treatment effect) and an *a priori*-postulated success rate for the control group (denoted $p_{C}^{\text{post}}$, a postulated success rate). Based on these postulated values, we calculated the required sample size, using a two-sided 5% type I error rate and a power of 80%. We used this sample size (based on postulated values) for the simulation study, but set the control arm success rate and treatment effect within each simulated trial based on the true parameter values (i.e., using the true parameters *θ* and *p*_*c*_). Finally, we analyzed the data by estimating the log of the odds ratio and a 95% confidence interval (95% CI).

In the situation where there was no treatment effect, we drew a treatment effect value *θ* from a normal distribution with mean 0 and success rate *p*_*c*_ from the Beta distribution. We then simulated data for a trial of sample size 300, and data were analyzed by estimating the log of the odds ratio and a 95% CI. Details of parameters for the distributions and calculations are in the S1 File.

Meta-experiment approach: in the meta-experiment approach, we neither *a priori*-specify any parameter nor perform a sample size calculation. Because our meta-experiment approach involves a meta-analysis of 3 randomized trials of fixed sample size of 100, the simulation algorithm is as follows: we drew 3 treatment effect values from the normal distribution of treatments effects– *θ*^1^, *θ*^2^ and *θ*^3^ –with 3 associated success rates for the control groups– $p_{C}^{1}{,\text{ p}}_{\text{C}}^{2}$ and $p_{\text{C}}^{3}$ –from the Beta distribution. Then, we simulated 3 randomized trials of size 100 each (i.e., 50 patients per group) with these parameters. Finally, we meta-analyzed the 3 estimated treatment effects. We used a random-effects model, allowing the estimated treatment effect to vary among the studies.

#### Simulation parameters

Treatment effect: we consider 2 distinct situations allowing for a treatment effect or not: OR of 1 (no treatment effect) and 1.5 (non-null treatment effect). Moreover, we assumed inter-study heterogeneity on the treatment effect [17] because of patient characteristics or how the intervention is implemented. Therefore, we defined a theoretical distribution for the true treatment effect, where the true effect is normally distributed, with mean = 0 in cases of no treatment effect and log(1.5) otherwise, with SD 0.1. The values were taken from a series of published meta-analyses [17,18].

Success rate in the control group: we also allowed the success rate associated with the control group to follow a probability distribution function. Indeed, patients may differ among studies, which may affect the theoretical success rate associated with the control group. Therefore, we used a Beta distribution, which allows the control arm success rate to vary between 0 to 100%, and set the mean to 30% with a SD of 10%.

#### Statistical outputs

We compared the statistical properties of the two approaches. We examined different statistical properties according to whether there was a treatment effect or not. Thus, for a non-null treatment effect, we assessed the following:

Power:

- the proportion of significant results
- the coverage rate defined as the proportion of runs with the true OR 1.5 within the estimated 95% CI

Precision:

- the width of the 95% CI for the estimated OR
- the number of patients included in the classical approach

For a null treatment effect, we assessed the following:

Type I error:

- the proportion of significant results

Precision:

- the width of the 95% CI for the OR

Computing details: for each approach, 10,000 simulations were run with use of R v2.15.2.

## Results

### Sample size

**Fig 2:**
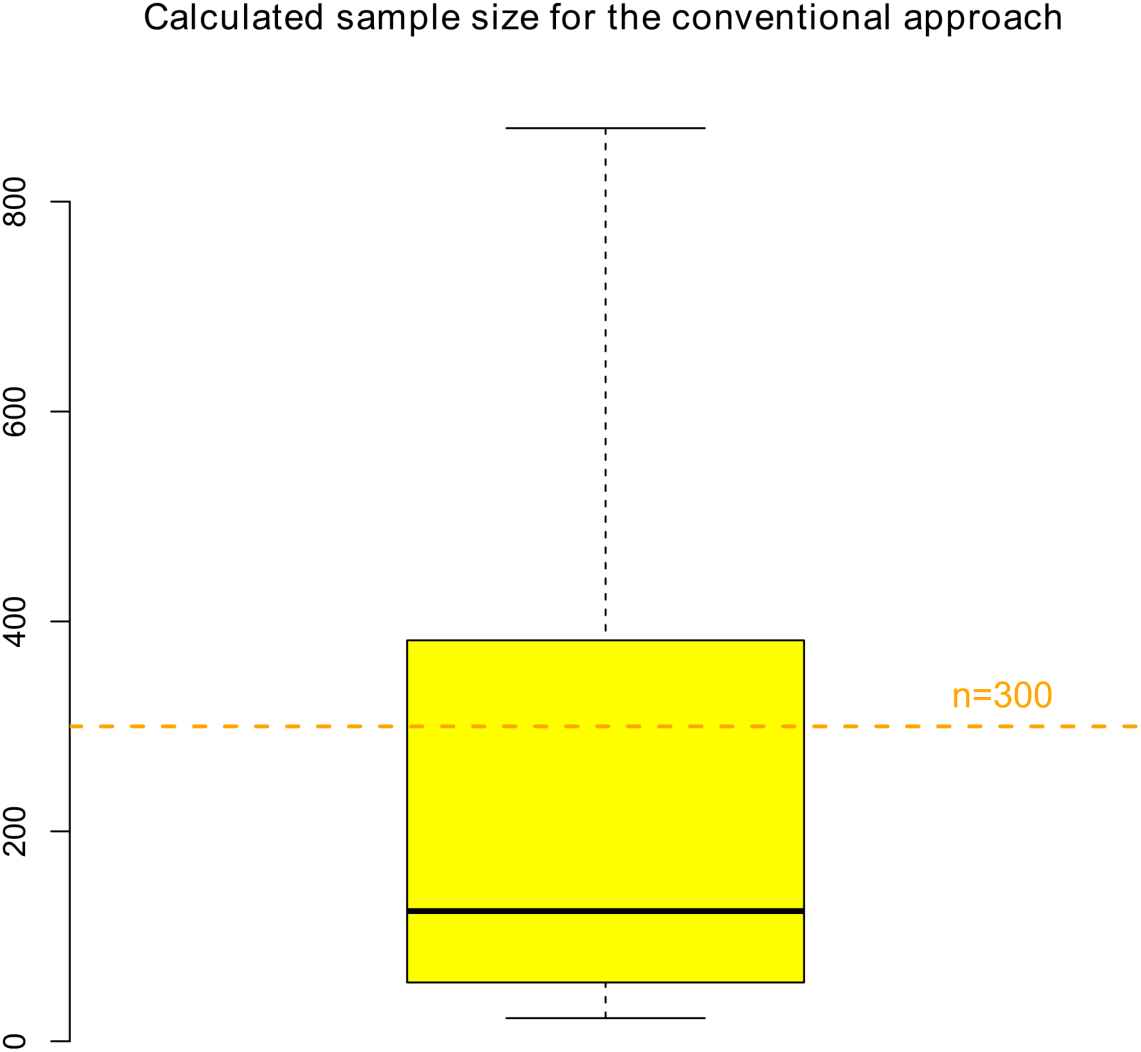
Box plot of the 10,000 calculated samples sizes based on the conventional approach. These sample sizes are used for the situation of mean theoretical odds ratio = 1.5. The dotted line represents 300 patients, which corresponds to the number of patients involved in the meta-experiment.

Calculated sample sizes for the classical approach for a non-null treatment effect are presented as a box plot in Fig 2. Theoretically, 1,100 patients is the required sample size for an odds ratio of 1.5 and a success rate of 30% in the control group. The median sample size is 122 (interquartile range 56–382; minimum 22, maximum 12,920). This distribution of sample sizes is consistent with the data from a previously published review that we used to calibrate the errors [6]. The mean sample size required with the classical approach is 443, so the statistical properties discussed hold in a situation in which the required number of patients is higher, on average, with the classical than the meta-experiment approach.

### Power and coverage rate

**Fig 3:**
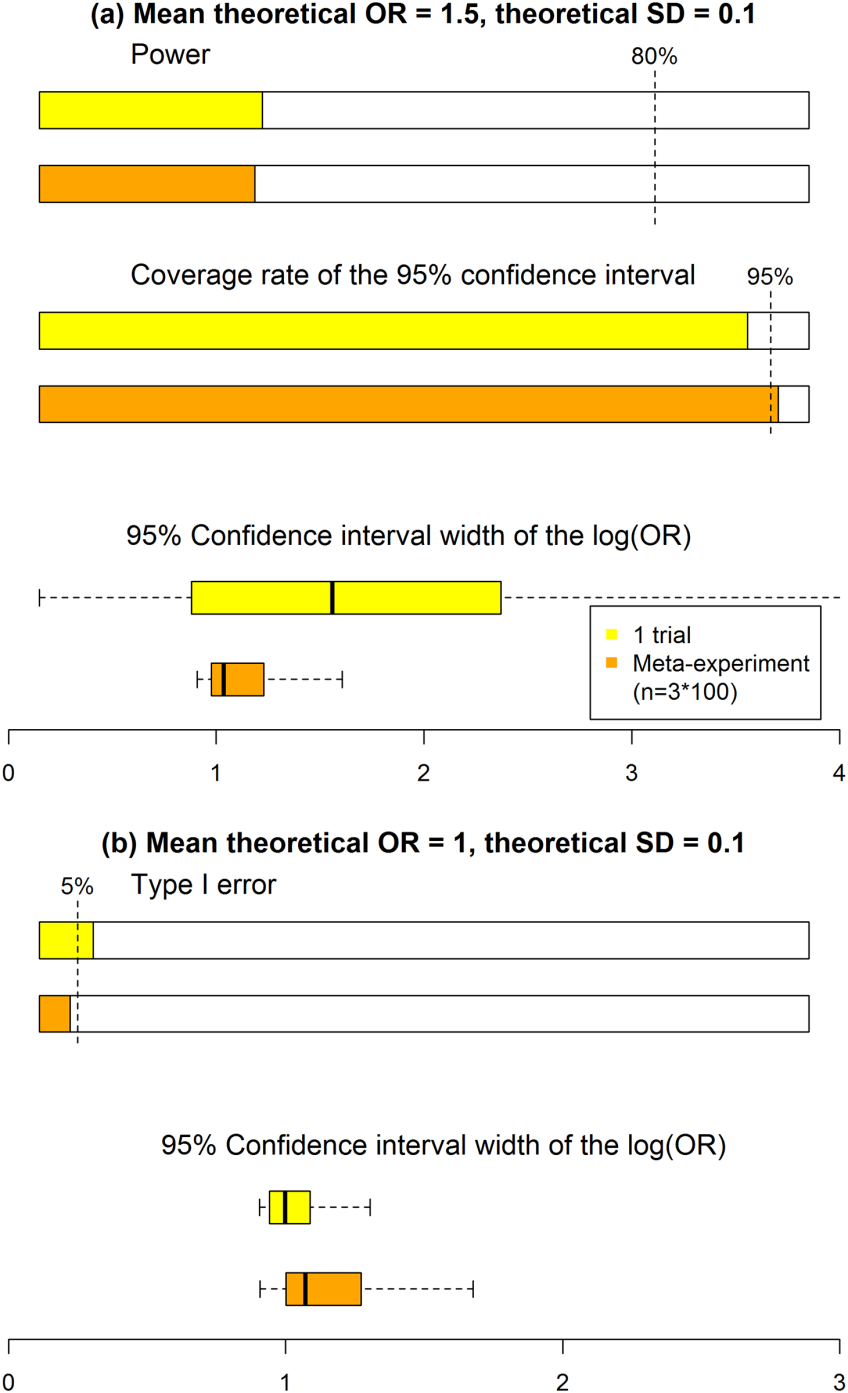
Statistical performance of conventional and meta-experiment approaches to determining sample size (1 trial vs 3 trials). (a) For a treatment effect (mean theoretical odds ratio (OR) = 1.5), power and coverage of the 95% confidence interval (95% CI) are estimated. (b) For no treatment effect (mean theoretical OR = 1), the type I error rate is estimated. For both scenarios, box plots of 95% CIs are presented. Results were derived from a simulation study with 10,000 runs for an expected mean success of 30% for the control group (i.e., considering a Beta distribution with mean 30% and SD 10%), and SD = 0.1 for the theoretical log OR.

Statistical properties are displayed in Fig 3. With a non-null treatment effect, the empirical power was nearly equal between the meta-experiment and classical approaches (28% and 29%). Thus, the rate of “qualitative” error (i.e., the inability to conclude a significant treatment effect, when it exists) is the same, whatever the approach. Both empirical powers are far from an 80% nominal value because of the median sample sizes of 122 and 300, while, as previously stated, 1,100 patients would have been required. Second, the coverage rate was slightly higher with the meta-experiment than classical approach (96% vs 92%). This result shows a better performance of the meta-experiment than classical approach in terms of “quantitative” errors. Third, the median 95% CI width was estimated at 1.04 for the meta-experiment as compared with 1.56 for the classical approach, so the meta-experiment approach leads to greater precision in the treatment effect estimate than the classical one.

### Type I error rate

With no treatment effect, the type I error rate was slightly higher with the classical than meta-experiment approach (7% vs 4%). The median 95% CI width was estimated at 1.07 for the meta-experiment as compared with 1.61 for the classical approach, which again shows better precision with the meta-experiment than classical approach.

We reran these simulations using relative risks instead of OR and obtained the same results. We reran these simulations for *θ*^th^ = log(2) (results in S1 Fig) and $\sigma_{\theta }^{\text{th}}=0.2$ (results in S2 Fig). The conclusions were similar. We reran these simulation for *θ*^th^ = log(1.5), $\sigma_{\theta }^{\text{th}}=0.1$, with 50 participants in each trial of the meta-experiment (results in S3 Fig) *θ*^th^ = log(2.5), $\sigma_{\theta }^{\text{th}}=0.1$, with 50 participants in each trial of the meta-experiment (results in S4 Fig) and *θ*^th^ = log(1.5), $\sigma_{\theta }^{\text{th}}=0.1$, with 200 participants in each trial of the meta-experiment (results in S5 Fig).

## Discussion

A prospective meta-analysis of data from trials of fixed sample size provided the same precision, power and type I error rate, on average, as the classical approach. From a statistical viewpoint, the conclusions drawn with the meta-experiment approach would be as valuable as those drawn with the classical single-trial approach, without the need for a greater number of patients on average. Despite a greater median sample size, the mean sample size is to be noted because it informs on the actual societal costs of research. Therefore, the meta-experiment efficiently addresses the sample size challenge. However, our results are based on a high success rate in the control group because the beta distribution we used led to 95% of simulated success rates between 12% and 51%.

The meta-experiment would also offer additional advantages. First, it would provide more complete information than does the classical approach. Indeed, a meta-analysis, besides synthesizing the effect size estimates, considers the aspect of replication. Replication is fundamental because an original study showing a statistically significant effect may be followed by subsequent studies reaching opposite conclusions or suggesting that the effect found in the original study was too strong [19]. Therefore, results from a single trial must be considered cautiously. Results being consistent across trials is a strong argument in favor of a robust treatment effect over different conditions. Otherwise, heterogeneity between studies must be reported and explored [20]. Indeed, Borm et al. [21] showed that heterogeneity may be a reason for performing several underpowered trials rather than a single large one.

A second advantage is that from a practical viewpoint, performing different independent trials across multiple countries may be easier than conducting a large multinational study and would reduce the duration and global cost of the study [22]. Indeed, clinical regulations, basically created to ensure patient safety, can have an adverse effect because they vary among countries, which can prevent the performance of multinational clinical trials [23]. This burden threatens especially academic trials, which often do not have adequate administrative support [24]. The meta-experiment approach would facilitate the performance of parallel trials in different countries. A consortium that has already adopted this approach is the Blood Pressure Lowering Treatment Trialists Collaboration, which has designed prospective meta-analyses [25,26]. This approach should become more common. The remaining difficulties are to fund 3 trials in 3 different countries and to convince researchers to collaborate internationally.

A remaining question is the appropriate way to go from there. If the meta-experiment has shown the existence of a relevant treatment effect, it has answered the question. However, if the meta-analysis cannot prove or exclude the existence of a relevant treatment effect, then more trials can be planned within the meta-experiment, and the meta-analysis is then updated. A meta-experiment can produce repeated analysis with each addition of new data. It should be noted that if the meta-analysis is simply reestimated with new trials, the 95% confidence interval of the treatment effect will not have the expected 95% coverage due to the former analysis. Therefore sequential methods are recommended to retain correct coverage rates even after several analyses [27].

## Conclusion

Prospectively planned meta-analyses could promote a collaborative culture, thus resulting in research that is more effective and less wasteful [15]. Meta-experiments would lead to the earlier benefit of effective interventions in practice, to reduce exposure of trial participants to less effective treatments and to reduce waste from unjustified research [28]. In addition, this approach avoids the publication bias that affects traditional systematic reviews [29] (i.e., the tendency for investigators, editors and reviewers to take into account trials’ results mainly on the basis of the unsound dichotomy of statistical significance).

## Supporting Information

S1 File Full algorithm. (PDF)

S1 Fig Scenario with higher mean theoretical treatment effect. (TIF)

S2 Fig Scenario with higher theoretical heterogeneity. (TIF)

S3 Fig Scenario with less participants in each trial. (TIF)

S4 Fig Scenario with less participants in each trial and higher mean theoretical treatment effect. (TIF)

S5 Fig Scenario with more participants in each trial. (TIF)
