# Supplementary material for: Finding Alternatives to the Dogma of Power Based Sample Size Calculation: Is a Fixed Sample Size Prospective Meta-Experiment a Potential Alternative?
Source: PLoS One. 2016 Jun 30;11(6):e0158604. doi: 10.1371/journal.pone.0158604 (PMC4928786; doi:10.1371/journal.pone.0158604)
Supplement: S1 File — (PDF) [file pone.0158604.s001.pdf]

1 Appendix

We assume that the true log odds ratio θ is normally distributed with mean μ_{theta}^{th} and standard deviation (SD) σ_{θ}^{th}. The success rate in the control group p_C is assumed to be beta distributed. The success rate in the treatment group p_T is deduced from p_C and θ.

Conventional approach

Theoretical values

For one simulation, the theoretical values are sampled from the theoretical distribution:

$$\theta \sim \mathcal{N}(\mu_{\theta}^{th}, \sigma_{\theta}^{2th}) \tag{1}$$

$$p_C \sim \mathcal{B}eta(6, 14) \tag{2}$$

In the situation with no treatment effect $\mu_{\theta}^{th} = 0$, in the situation with a non null treatment effect $\mu_{\theta}^{th} = log(1.5)$. The SD σ_{θ}^{th} equals 0.1. p_T is calculated from the formula

$$\frac{p_T/(1-p_T)}{p_C/(1-p_C)} = \exp(\theta) \tag{3}$$

Postulated hypothesis

We then consider that the postulated hypothesis used for the sample size calculation differs from the theoretical values. We let ϵ_{theta} be the relative error on the log OR and ϵ_{p_C} the relative error on the control group event rate after an angular transformation. We used data from a previously published review [1] to calibrate these errors: they are sampled from normal distributions with mean 0.082 and SD 5.583 for ϵ_{theta} and mean 0.026 and SD 0.301 for ϵ_{p_C}.

$$\theta^{post} = (1 + \epsilon_{theta})\theta \tag{4}$$

$$arcsin(\sqrt{p_C^{post}}) = (1 + \epsilon_{p_C})arcsin(\sqrt{p_C}) \tag{5}$$

p_T^{post} is calculated from the formula

$$\frac{p_T^{post}/(1-p_T^{post})}{p_C^{post}/(1-p_C^{post})} = \exp(\theta^{post}) \tag{6}$$

Furthermore, the success rates are restricted to [5%; 95%] and the treatment effect is restricted to [0.2;2.22], the 95% range observed on the data.

Sample size calculation

The number of patients to be recruited in each group is calculated as:

$$n = \frac{2(Z_{1-\frac{\alpha}{2}} + Z_\beta)^2}{\Delta^2} \tag{7}$$

with

$$\Delta = \frac{p_T^{post} - p_C^{post}}{\sqrt{\frac{p_T^{post}(1-p_T^{post})+p_C^{post}(1-p_C^{post})}{2}}}, \tag{8}$$

and Z_q is the q-percentile of a standard normal distribution.

Data generation

The number of events in the control group X_C^n and the treatment group X_T^n are sampled from binomial distributions. The number of events are sampled considering the true parameter p_C and p_T rather than the postulated ones, p_C^{post} and p_T^{post}.

$$X_C^n \sim \mathcal{B}(n, p_C),\ X_T^n \sim \mathcal{B}(n, p_T), \tag{9}$$

The observed treatment effect is the log odd ratio $\hat{\theta}$ defined as

$$\hat{\theta} = log\left(\frac{x_T^n/(n - x_T^n)}{x_C^n/(n - x_C^n)}\right), \tag{10}$$

with estimated variance V

$$V = 1/x_C^n + 1/(n - x_C^n) + 1/x_T^n + 1/(n - x_T^n). \tag{11}$$

Analysis

The 95% confidence interval for the log odd ratio is calculated as

$$95\%\ \text{CI} = [\hat{\theta} - Z_{1-\frac{\alpha}{2}}\sqrt{V}; \hat{\theta} + Z_{1-\frac{\alpha}{2}}\sqrt{V}] \tag{12}$$

Meta-experiment approach

The meta-experiment approach involves three trials of 100 subjects each.

Theoretical values

For one simulation, the 3 sets of theoretical values are sampled from the theoretical distributions:

$$\theta^i \sim \mathcal{N}(\mu_\theta^{th}, \sigma_\theta^{2th}), i = 1, 2, 3 \tag{13}$$

$$p_C^i \sim \mathcal{B}eta(6, 14), i = 1, 2, 3 \tag{14}$$

p_T^i are calculated from the formula

$$\frac{p_T^i/(1 - p_T^i)}{p_C^i/(1 - p_C^i)} = \exp(\theta^i), i = 1, 2, 3 \tag{15}$$

Sample size calculation

The number of subjects to be recruited in each trial is 100.

Data generation

For trial $i = 1, 2, 3$, the number of events in the control group X_C^i and the treatment group X_T^i are sampled from binomial distributions

$$X_C^i \sim \mathcal{B}(50, p_C^{th,i}),\ X_T^i \sim \mathcal{B}(50, p_T^{th,i}), \tag{16}$$

and the observed treatment effects are the log odds ratios $\hat{\theta}^i$ defined as

$$\hat{\theta}^i = log\left(\frac{x_T^i/(50 - x_T^i)}{x_C^i/(50 - x_C^i)}\right), \tag{17}$$

with estimated variance V_i

$$V^i = 1/x_C^i + 1/(50 - x_C^i) + 1/x_T^i + 1/(50 - x_T^i). \tag{18}$$

Analysis

The observed effect $\hat{\theta}^i$ for any study is given by the mean μ, the deviation of the study's true effects from the mean, and the deviation of the study's observed effect from the study's true effect. That is

$$\hat{\theta}^i = \mu + \xi^i + \epsilon^i, \tag{19}$$

with $\xi^i \sim \mathcal{N}(0, \sigma_\theta^{2th})$ and $\epsilon^i \sim \mathcal{N}(0, V^i)$. Under the random effect model the weight assigned to each study is $w^i = \frac{1}{V^i + \tau^2}$ where τ^2 is an estimate of σ_θ^{2th} from the existing studies (eg the DerSimonian-Laird estimate [2]). The meta-analysis effect estimate is

$$\hat{\theta} = \frac{\sum_{i=1}^{3} w^i \hat{\theta}^i}{\sum_{i=1}^{3} w^i} \tag{20}$$

with approximate variance

$$var(\hat{\theta}) = \frac{1}{\sum_{i=1}^{3} w^i} \tag{21}$$

The 95% confidence interval for the log odd ratio is calculated as

$$95\%\ \text{CI} = [\hat{\theta} - Z_{1-\frac{\alpha}{2}}\sqrt{var(\hat{\theta})}; \hat{\theta} + Z_{1-\frac{\alpha}{2}}\sqrt{var(\hat{\theta})}] \tag{22}$$

References

[1] Charles P, Giraudeau B, Dechartres A, Baron G, Ravaud P. Reporting of sample size calculation in randomised controlled trials: review. BMJ (Clinical research ed). 2009;338:b1732.

[2] DerSimonian R, Laird N. Meta-analysis in clinical trials. Controlled Clinical Trials. 1986 Sep;7(3):177–188.
