# Supplementary figures and images for: Finding Alternatives to the Dogma of Power Based Sample Size Calculation: Is a Fixed Sample Size Prospective Meta-Experiment a Potential Alternative?
Source: PLoS One. 2016 Jun 30;11(6):e0158604. doi: 10.1371/journal.pone.0158604 (PMC4928786; doi:10.1371/journal.pone.0158604)

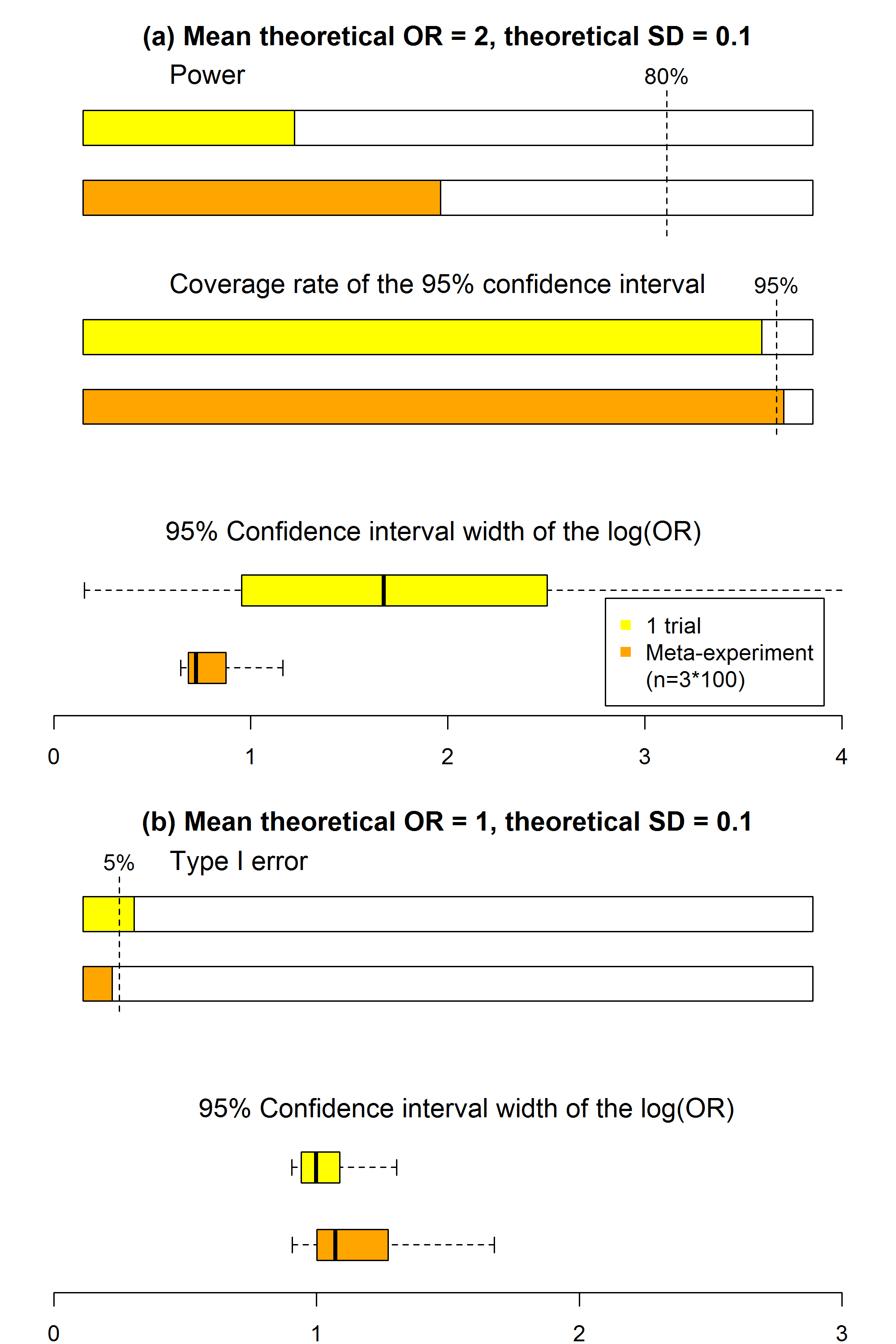

Supplement: S1 Fig — (TIF) [file pone.0158604.s002.tif]

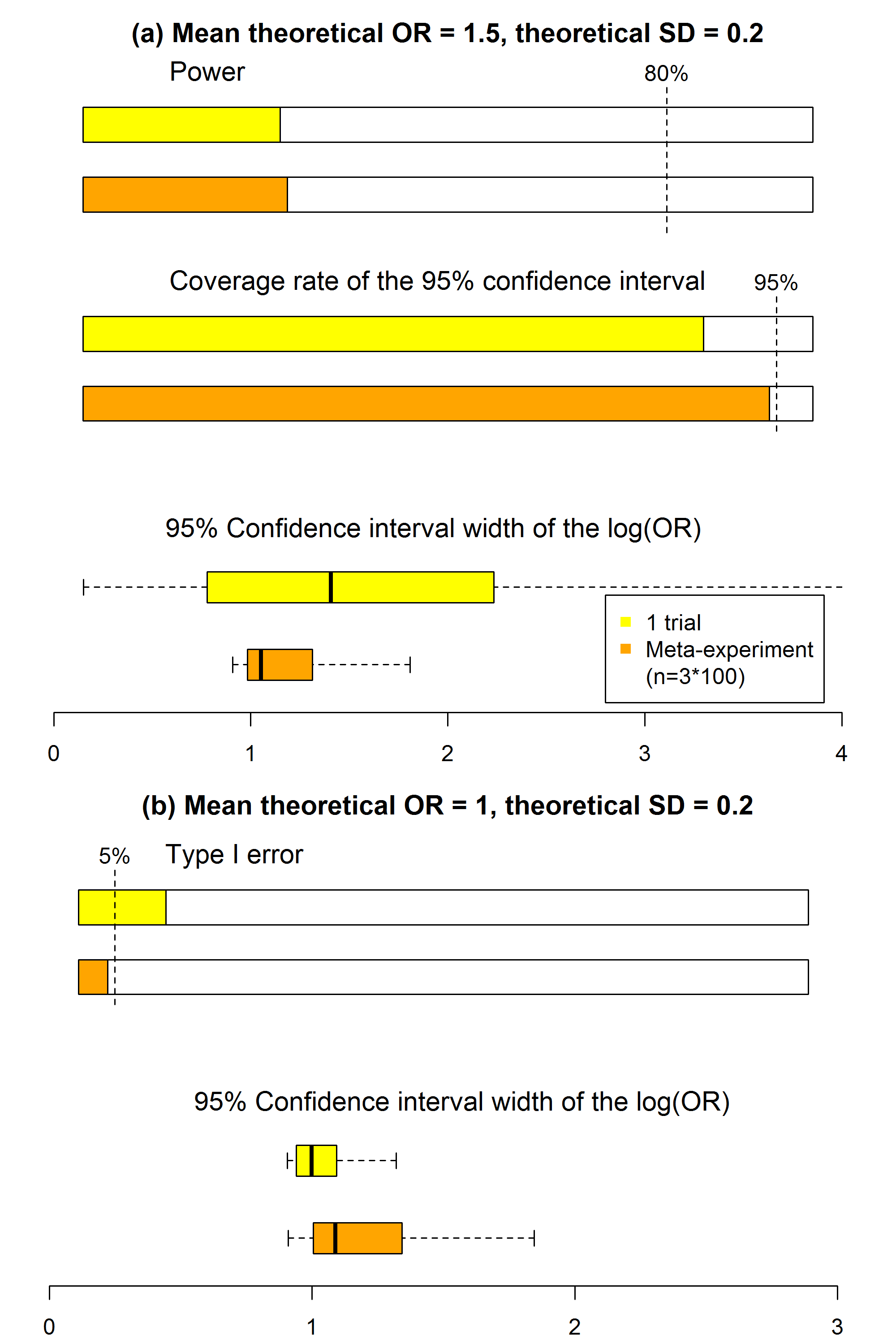

Supplement: S2 Fig — (TIF) [file pone.0158604.s003.tif]

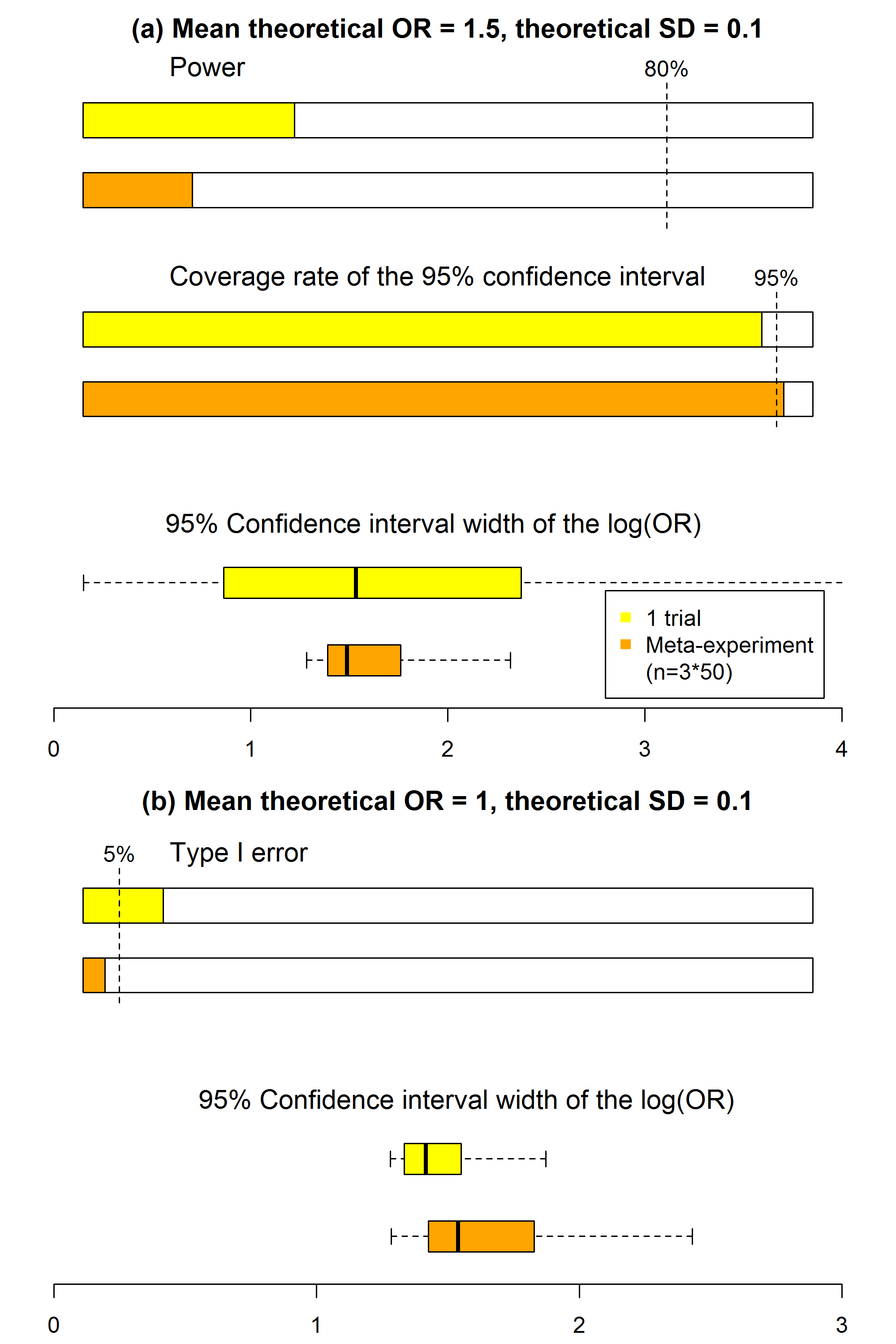

Supplement: S3 Fig — (TIF) [file pone.0158604.s004.tif]

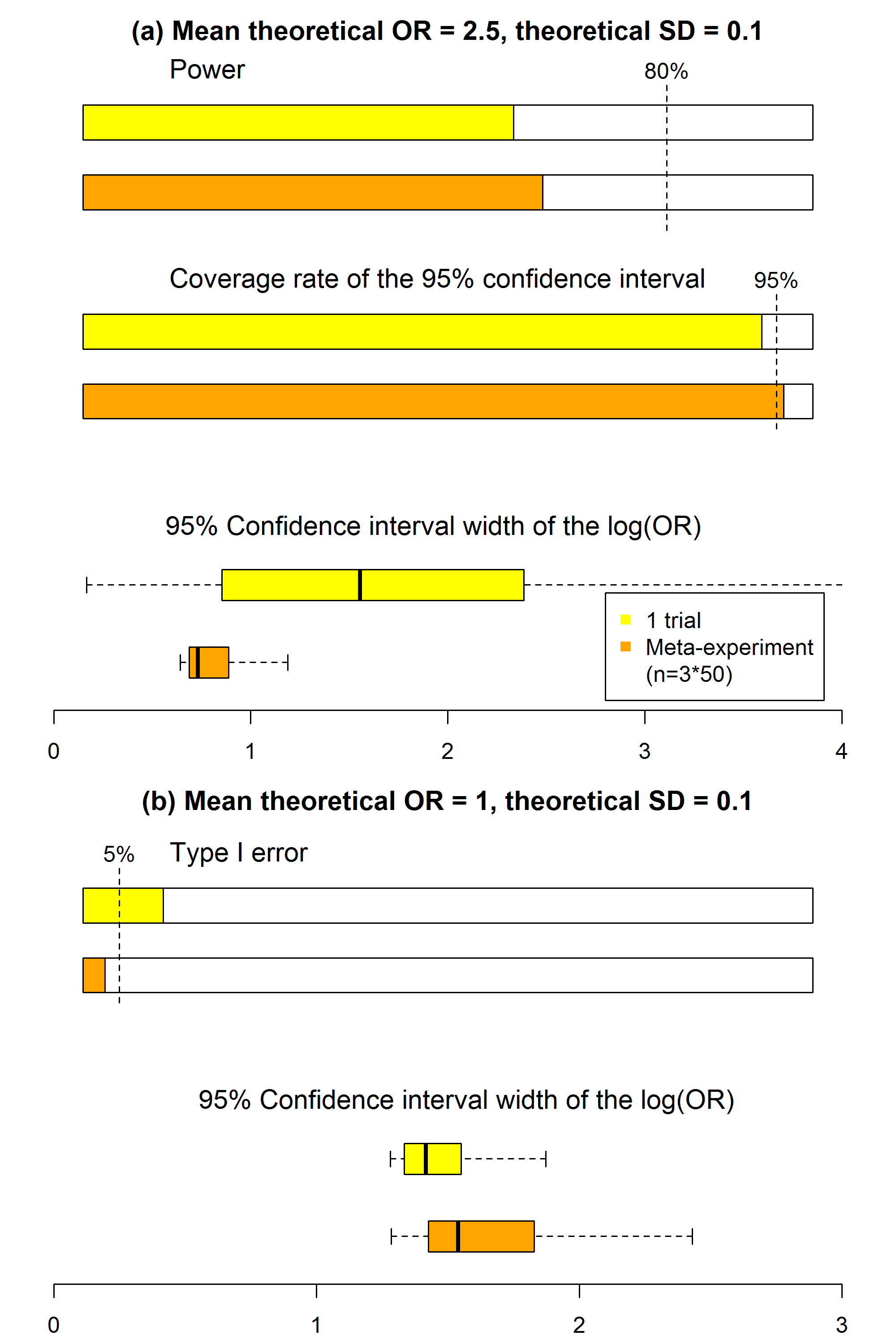

Supplement: S4 Fig — (TIF) [file pone.0158604.s005.tif]

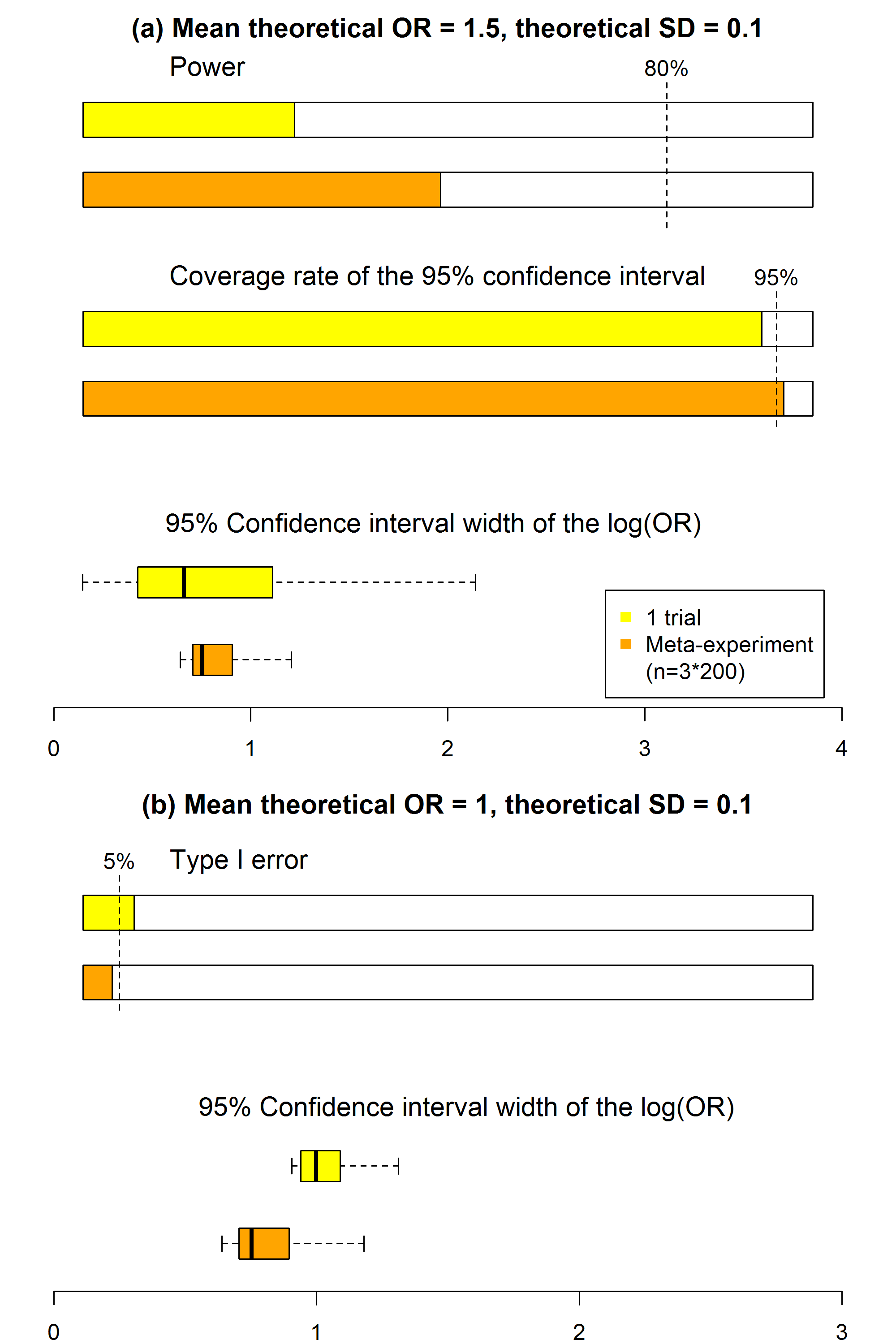

Supplement: S5 Fig — (TIF) [file pone.0158604.s006.tif]
